# Rapid increase of overweight and obesity among primary school-aged children in the Caribbean; high initial BMI is the most significant predictor

**DOI:** 10.1186/s40608-018-0182-8

**Published:** 2018-01-30

**Authors:** Walaa A. Mumena, Isabella Francis-Granderson, Leroy E. Phillip, Katherine Gray-Donald

**Affiliations:** 1 Clinical Nutrition Department, College of Applied Medical Sciences, Taibah University, Al-Madinah, Saudi Arabia; 2 Faculty of Food and Agriculture, University of the West Indies, St. Augustine Campus, Trinidad and Tobago; 3 Department of Animal Science, McGill University 21, 111 Lakeshore Road, Sainte-Anne-de-Bellevue, Québec H9X 3V9 Canada; 4 School of Dietetics and Human Nutrition, McGill University 21, 111 Lakeshore Road, Sainte-Anne-de-Bellevue, Québec H9X 3V9 Canada

**Keywords:** Obesity, Incidence, Predictors, Caribbean children, Misreporting

## Abstract

**Background:**

To examine predictors of increasing overweight among children in two developing countries.

**Methods:**

Primary school children (6–10 y at baseline, *n* = 336) and their caregivers.

Longitudinal data were collected in 2012, with follow-up 18 months later. Data on children’s height, weight and dietary intake were collected within 8 primary public schools in Trinidad and 7 schools in St. Kitts. Caregivers’ demographic and anthropometric data were also collected.

**Results:**

At baseline, children’s age and sex and caregivers’ BMI, age, and marital status and reported dietary intake were similar across all weight groups. The incidence of overweight and obesity among children was 8.8% and 8.1%, respectively. Dietary intake at baseline was not related to becoming overweight or obese. Similarly there were no differences in reported intake among children who became overweight or obese except that they consumed fewer fruits (0.54±0.92 vs. 0.98±1.66, *p* = 0.017). Misreporting of energy intake was higher among overweight/obese children as compared to those who were not overweight/obese (27% vs. 17%, *p* = 0.047). The baseline predictors of increasing BMI (adjusted) of the children were older age, higher baseline BMI z-score and higher height-for-age (HFA) z-score; caregiver BMI, children’s energy intake (with adjustment for misreporting) did not predict changes in children’s BMI.

**Conclusions:**

The increasing prevalence of overweight/obesity among children is a serious problem in the Caribbean. Heavier children are at elevated risk of continued rapid increase in their weight status, pointing to the need for early intervention.

## Background

The prevalence of obesity has increased over the last few decades and has now become a worldwide public health problem [1]. It is becoming more prevalent among all age groups including children in developing countries [2]. Childhood obesity is a matter of concern because obese children are at higher risk of becoming obese adults [3–5], and are at increased risk of suffering from non-communicable chronic diseases, such as diabetes and coronary heart disease, later in life [6]. Moreover, obese children already have a higher prevalence of insulin resistance [7, 8] and elevated blood pressure [9].

Predictors of overweight and obesity among children in the developed world are well investigated, but this is not the case in developing countries. Energy intake, as well as certain dietary patterns [10–13], low levels of physical activity [14, 15], high baseline BMI [15] and parental BMI [15–17] are well-known predictors of weight gain among populations in developed countries. The “nutrition transition”, defined as the shift to more “Westernized” dietary practices and increasingly sedentary lifestyles, has been linked to the increased prevalence of childhood overweight and obesity in developing countries [18, 19]. In the Caribbean, the prevalence of overweight and obesity among children is steadily increasing. Between 1990 and 1999, childhood obesity in Dominica increased from 6.0% to 9.7%, while in St. Kitts the prevalence has increased from 7.1% to 10.6% [20]. A recent study in Barbados, using identical reference standards, reveal that between 1981 and 2010 the prevalence of overweight and obesity among children aged 8–11 years (*n* = 580) increased from 8.5% to 32.5% [21].

The purpose of this study was to determine the incidence of becoming overweight or obese over a period of 18 months for children aged 6 to 10 years living in two Caribbean countries, and to examine dietary and other predictors of increasing adiposity for these children.

## Methods

This study was part of a broader multidisciplinary project dealing with food and nutrition security in the Caribbean Community (CARICOM), with a focus on improving nutritional outcomes of children in the region; details of the broader project work reported elsewhere [22]. Ethical approvals were obtained from the McGill Research Ethics Committee; the Ministry of Education in St. Kitts and Nevis; the Ministry of Health St. Kitts and Nevis; the Ministry of Food Production, Land and Marine Affairs in Trinidad and Tobaggo; the Ministry of Health, and the Ministry of Education, Republic of Trinidad and Tobago, Research and Publication Unit.

Data for this study were collected between January and July 2012 in a first phase and follow-up data were collected between September 2013 and April 2014. Children aged 6–10 years and their caregivers were recruited from eight schools in Trinidad and Tobago and seven schools in St. Kitts and Nevis. The population in Trinidad and Tobago is a mix of Afro-Caribbean and Indo-Caribbean peoples, while in St. Kitts and Nevis the population is solely Afro-Caribbean. In Trinidad, schools were selected from those with a high proportion of children who received a government-provided school lunch and included both rural and urban schools. Schools in St. Kitts, where all children are offered a free lunch, were selected from rural areas near the capital of Basseterre. A letter to parents requesting the participation of caregiver and their child was provided to the household and only those children with signed parental consent were enrolled. For families with more than one child in the desired age range, one child was randomly chosen. Interviews were conducted at home and trained interviewers collected a single 24-h dietary recall for each child, undertaken with the child and caregiver together as recommended [23]. Demographic data collected included caregiver’s age, sex, marital status, education and household size; measured height and weight of the caregiver were recorded during the home interview, and children’s height and weight were measured at school.

Height was measured as “standing height” using a stadiometer, and body weight was measured using a digital floor scale. Weight of children was measured with no shoes, light clothing and empty pockets. BMI z-score following the World Health Organization (WHO) reference [24] was used to classify children’s weight status. Change in children’s BMI was calculated as suggested by Cole et al. [25] to avoid the ceiling effect of high percentiles and modeled z-scores. These were calculated by subtracting the sex–age-specific median BMI using the WHO reference from the measured BMI from baseline and follow-up measures. The median BMI for a child of 6 years is 15.3 for both sexes and for 12 year olds it is 18.0 for girls and 17.5 for boys. The change in median BMI over 18 months is very small from 6 to 7.5 years (0.2–0.3) and from 11 to 12.5 it is higher 0.8 for girls and 0.6 for boys.

To assess children’s growth, which was a concern in the Caribbean region, height-for-age z-score (HFA z-score) was calculated using the WHO reference at each time point [26]. As an additional outcome measure to the adjusted change in BMI, a child was considered an incident case of overweight or obesity when a healthy weight child became overweight or obese, and when an overweight child became obese.

One 24-h dietary recall was conducted at baseline by locally trained interviewers. Caregivers and their children were interviewed at home and they were asked to provide details on the types and amounts of foods that were consumed by the child on the previous day (including vitamin and mineral supplements). Home interviews were done on different days of the week including weekends and holidays providing a sample of higher between-person variation of food intake. Data for a number of children were collected the day after a non-school day and holidays. Portion models (Santé Quebec, Montreal, Canada) were used to help in the estimation of amounts consumed in milliliters; values for each food item were then converted to grams for data entry. CANDAT Nutrient Analysis Software (Godin London Incorporated, London, ON), was used for the nutrient analysis based on the Canadian Nutrition Files released in 2010 [27]. Where processed foods were locally produced, food labels or local recipes were added to the database. Foods groups were formed based on the Six Caribbean Food Groups (staples, legumes and nuts, foods from animals, fruits, vegetables, fats and oils) [28]. Green banana, plantain, breadfruit, corn, sweet potato and cassava are staples based on the Caribbean Food Groups, and serving sizes for these foods were calculated based on average carbohydrate content in one serving of grain. Portions of all other foods followed Canada’s Food Guide portion sizes [29]. As the mean intake of a single day is a reliable measure of the intake of groups [30], this was used to compare the dietary intake of children who became overweight or obese versus children who remained at the same weight category. Food groupings were not used in regression models as the foods eaten by each child on 1 day is not representative of usual intake for a specific child for a specific food group.

To examine the extent of misreporting of energy intake and to adjust for it in the analysis, the ratio of reported energy intake (EI) to estimated total energy expenditure (TEE) was used to classify children to the appropriate misclassification group based on Goldberg cut-offs. Total energy expenditure was determined for each child using Torun equations [31]. A detailed explanation of this method is available elsewhere [32]. Children were classified as either “under-reporters”, defined as having a ratio of EI:TEE < 0.76, “plausible-reporters”, having ratio of EI:TEE between 0.76 and 1.24, and “over-reporters”, having ratio of > 1.24 [32].

Descriptive data for the characteristics of children and their caregivers are presented as mean ± SD, and categorical variables are presented as percentages. WHO cut-off points were used to define caregivers weight status: thinness < 18.5 (kg/m^2^), healthy weight 18.5–24.9 (kg/m^2^), overweight 25–29.9 (kg/m^2^), obesity ≥30 (kg/m^2^) [33]. The WHO cut-off points for BMI z-score to define children’s weight status are (severe thinness, < − 3 (SD); thinness, < − 2 (SD); overweight, > 1 (SD); and obesity, > 2 (SD)) [24]. The chi-square test was used to compare categorical data, while the t-test was used to compare mean for two groups and to compare means for 3 groups or more, ANOVA was used. Multiple regression analysis was performed to test for association between baseline BMI z-score, baseline HFA z-score, age, energy intake, misreporting of energy intake (under-reporters= −1, plausible-reporters = 0, over-reporters = 1), and caregiver BMI in relation to the change of children’s BMI. All statistical tests were 2-tailed, and a significance level of *p* < 0.05 was adopted. Correcting for multiple comparisons (dietary intake and weight category) was conducted using the Bonferroni method (dietary intake: *p* < 0.006 and food groups: *p* < 0.01). All statistical analyses were performed using SAS® software version 9.4 (2013, SAS Institute Inc., Cary, NC, USA).

## Results

A total of 336 children and their caregivers were included in this study. We excluded 77 children (15.6%) due to missing dietary data at baseline or follow up as appointments could not be conducted or caregivers’ refusal to collect dietary data and 78 children (15.7%) were excluded due to missing anthropometric data (moved to different school or were absent in the day of data collection). We also excluded 3 children (0.60%) due to very low < 700 (kcal) or high > 3800 (kcal) reported energy intake.

Demographic data for children and caregivers at baseline by weight categories of the children are shown in Table 1. The caregivers were mostly female (94%) and overweight or obese (mean BMI 40.1±9.08 (kg/m^2^)). Caregivers were similar in age, sex and marital status across all children’s weight categories at baseline. There were no differences in children’s age or sex among the all weight categories of the children.

**Table 1:** Baseline demographic characteristics of children and their caregivers by children’s baseline weight status in two Caribbean countries, St. Kitts and Nevis and Trinidad and Tobago (*n* = 336)

	Thin (*n* = 14)	Healthy weight (*n* = 248)	Overweight (*n* = 45)	Obese (*n* = 29)
Children				
Age, mean±SD	7.3±0.82	7.4±0.94	7.3±0.92	7.5±0.95
Sex, female, %	14.3	48.8	48.9	48.3
HFA z-score, mean±SD	-0.12±0.69	0.26±0.91	0.99±0.91	1.12±1.01
BMI z-score, mean±SD	−2.55±0.51	−0.39±0.71	1.39±0.32	2.90±0.84
Caregiver				
Age, mean±SD	35.6±10.4	34.5±9.5	34.8±9.1	39.6±10.4
Sex, female, %	93.3	95.4	98.7	96.6
Unmarried, %	57.1	46.2	57.6	69.2
BMI, mean±SD	36.9±6.9	40.1±9.5	40.1±8.3	41.9±6.9
Country,				
Trinidad %	86.0	52.4	57.8	58.6
Education				
< secondary, %	43.8	43.8	52.5	35.3

*SD* standard deviation

A comparison of demographic variables, dietary intake and anthropometric measures at baseline and follow-up between countries indicated no differences were found. Results of children’s weight status at baseline and at follow-up are shown in Table 2. At baseline, 22.0% of children were overweight or obese, while at follow-up 28.6% were overweight or obese. At follow-up, the percentage of children in the healthy weight category decreased by 6.8%, while the percentage of overweight children remained the same and the percentage of obese children increased by 6.9%. The incidence of healthy weight children becoming overweight was 8.8% and healthy weight and overweight children becoming obese was 8.1%. A total of 15.1% (*n* = 48) of children moved up to a higher weight category to become either overweight or obese, while only 1.58% (*n* = 5) children moved down a weight category. The percentage of thin children (BMI z-score < − 2 (SD) remained constant.

**Table 2:** Children’s weight status at baseline and follow-up in two Caribbean countries, St. Kitts and Nevis and Trinidad and Tobago (*n* = 336)

	Thin	Healthy weight	Overweight	Obese	*p*
Baseline	14 (4.2)	248 (73.8)	45 (13.4)	29 (8.6)	< 0.001
Follow-up	15 (4.5)	225 (67.0)	44 (13.1)	52 (15.5)	

Chi-square test was preformed

Absolute values (*n*) are provided in the table; values in parenthesis represent percentages

Baseline dietary intakes of children were similar across the different baseline weight categories. The dietary intake of children who become overweight or obese was generally similar to the intake of children who did not move up a weight category (excluding thin children and children who were obese at baseline). The one exception was that children who moved up a weight category tended to consume less servings of fruit. There was no difference in reported energy intake for those who moved into a higher weight category vs. those who did not. See Table 3.

**Table 3:** Baseline dietary intake by change in weight category of school-aged children 6–12 years from two Caribbean countries, St. Kitts and Nevis and Trinidad and Tobago (*n* = 296)^*^

	Children who did not move up a weight category (*n* = 252)	Children who became overweight or obese (*n* = 44)	*p*
Energy, kcal	1825±547	1781±536	0.628
Carbohydrate, g	275±96.2	263±99.0	0.447
Protein, g	59.0±26.3	62.6±21.4	0.269
Fat, g	57.1±26.7	57.4±23.9	0.813
Total sugar, g	113±59.4	104±61.0	0.283
Fiber, g	14.2±7.58	14.2±7.40	0.998
Calcium, mg	608±328	612±323	0.924
Iron, mg	13.7±9.40	14.1±9.50	0.984
Staple, portions	8.23±5.43	7.25±3.99	0.358
Foods from animals, portions	3.97±3.43	4.57±3.13	0.202
Legumes and nuts, portions	0.19±0.56	0.14±0.40	0.633
Fruits, portions	0.98±1.66	0.54±0.92	0.017
Vegetables, portions	1.10±1.67	1.19±1.30	0.206

^*^Thin children and children who were obese at baseline were excluded

Values presented are means ± standard deviations

As it is important to measure the magnitude of gain in relation to baseline weight, we examined the change in BMI for each quartile of baseline BMI z-score. The average BMI change in the lowest BMI quartile at baseline was positive at 0.68 of a BMI unit adjusted for age and sex. The gain in the children in the highest quartile at baseline was considerably higher at 2.5 BMI units (*p* < 0.001), (see Table 4). This increase in BMI units translates to an average weight gain of 12 (kg) (mean weight 32 (kg) at baseline and 44 (kg) at follow-up) for a child in the heaviest quartile; this can be compared to an average weight gain of 5 (kg) (25 to 30 (kg) if the child gained weight following changes expected at the median BMI for age and sex.

**Table 4:** Change in BMI among children by baseline quartiles of BMI z-score in two Caribbean countries, St. Kitts and Nevis and Trinidad and Tobago (*n* = 336)

	1st quartile	2nd quartile	3rd quartile	4th quartile	*p*
Number of children	83	85	85	83	<0.001
Change in BMI (adjusted^*^)	0.68±1.3^a^	1.1±1.1^b^	1.2±1.5^c^	2.5±1.6^d^	

Numbers are means ± standard deviations

^*^Adjustment for age was done by subtracting the sex–age-specific median BMI

^a, b, c, d^different superscripts indicate statistically different means

In multiple regression analysis, higher baseline BMI z-scores, higher baseline HFA z-scores and older age of children predicted increased change in BMI over the 18 months of follow-up. For each additional unit of baseline BMI z-score, the BMI increased by 0.43 BMI units (adjusted for age and sex), indicating that the heaviest children at baseline gained the most weight. This analysis was adjusted for energy intake, and the misreporting of energy intake as well as caregiver BMI but none of these variables were associated with increasing BMI over time. Misreporting of energy intake was evaluated to verify the validity of the dietary data. More under-reporting was found among overweight and obese children when compared to those children who were not overweight or obese (27% vs. 17%, *p* = 0.047); hence, we adjusted for misreporting in our regression model. The model is presented in Table 5.

**Table 5:** Multiple regression model of association with change in children’s BMI* from two Caribbean countries, St. Kitts and Nevis and Trinidad and Tobago (*n* = 336)

	Beta estimate	SE	*p*
Age, years	0.19	0.08	0.019
Baseline BMI z-score	0.43	0.06	< 0.001
Baseline HFA z-score	0.21	0.08	0.007
Energy intake, baseline, 100 kcal	−0.03	0.01	0.057
Misreporting of energy intake	0.16	0.24	0.499
Caregiver BMI	0.01	0.01	0.183
R-square	0.23		

*SE* standard error

*Adjustment for age was done by subtracting the sex-age-specific median BMI

## Discussion

In the present study we investigated the development of overweight and obesity among school-aged children in the Caribbean over 18 months and predictors of increasing BMI over time. There were important increases in overweight and obesity over the study period with 15% becoming overweight or obese vs. less than 2% of overweight/obese children getting into a healthier weight category. There were no differences in dietary intake among children in different weight categories at baseline, except a lower consumption of fruit was evident in those who increased a weight category. The major predictor of gains in BMI was the baseline BMI z-score, indicating that heavier children are those most likely to become even heavier.

The rapid increase in the prevalence of overweight and obesity among our sample of children was reflected by the mean change in BMI (adjusted for age and sex), which was 1.3 kg/m^2^. This increase above the WHO standard for growth at this age was less than observed in children aged 8–10 years in Québec (1.7 kg/m^2^). Both studies investigated changes in adiposity among children whose parents were overweight or obese [34]. Lower mean weight gains among children in our sample could be due to the fact that children in the Quebec study had a higher baseline level of overweight and the sample was slightly older. In this study, 9% of children increased their weight categories to become overweight and 8.1% became obese over the study period. These values are high when compared to findings from an Australian cohort study conducted over 3 years (1997–2000) with children aged 5–10 years at baseline (*n* = 1438); in that study, the incidence of overweight was similar (9.7%) but that for obesity was much lower (1.7%) [35].

Increased prevalence of overweight and obesity indicate an imbalance of EI and physical activity at the population level [14]. In this study, energy intake was similar across all weight categories. In fact, total energy expenditure was not investigated, but it was evident that overweight and obese children under-reported their energy intake compared to children who were normal weight. Misreporting of dietary intake data is a major challenge in gaining a better understanding of the foods actually consumed. Adjusting for the misreporting of energy intake when analyzing the data in order to establish more accurate epidemiological relationships is suggested [36], but the problem of knowing which foods are misreported remains a challenge and may lead to failing to pick up some types of foods that increase the risk of excessive weight gain.

Dietary intakes were similar among children from all weight categories and children who become overweight or obese versus children who did not; only fruit intake was lower among children who moved up weight category compared to children who did not. The relationship between fruit and/or vegetable intake and weight gain is evident among adults but among children the findings have been inconsistent, with some but not all showing a protective effect of fruits and vegetables [37].

In this study, children with higher weight at baseline gained the most weight over 18 months of follow-up and this is in line with findings of other studies. It has been reported in North America that heavier children are gaining weight more quickly than children with healthy weight children [15, 38] and reports indicate that approximately 50% of obese school-aged children will become obese adults [5]. In this study, height and age were also strong predictors for increased adiposity and that is in line with previous findings [39, 40].

Previous research has identified a positive association between parental BMI and children’s weight status [16, 17]. In this study, however, 97% of the caregivers were identified as overweight or obese, in the Caribbean young women have a higher prevalence of obesity than men [41]. The sex of the child was not a concern in this study as weight gain peaks for both girls and boys after the age of 12 years (for girls 12.5 years and for boys 14 years) [42, 43], and no sex difference is evident before this age.

The major strengths of this study include its longitudinal design, which allowed us to measure the change in BMI among children ages 6–10 years and not just examine secular changes over time. In addition, misreporting of energy intake was assessed in our study and controlled for in the regression analysis. Energy intake alone should not be used to measure energy balance unless misreporting is accounted for. However, some limitations of this study should also be considered. Our sample is not representative of the general population in the Caribbean because the schools in Trinidad were chosen based on their having a high proportion of children from families considered to be “in need”. Also, reliable measures of physical activity were not available so that one cannot know if the important weight gains in the heavier children were due to lower levels of physical activity in the heavier children.

## Conclusions

The prevalence of overweight and obesity is known to be a rapidly growing problem among children in the Caribbean. Predictors of excessive weight gain in individual primary school children clearly show that the heaviest children gain the most excess weight over a relatively short period of time, thereby adding to their health burden. Interventions early in a child’s life to lessen the risk of becoming overweight and to help overweight and obese children to not continue to gain excessive weight are important to reduce non-communicable diseases in the future.

## Abbreviations

BMI: Body Mass Index
CARICOM: Caribbean Community and Common Market
EI: Reported energy intake
HFA: Height-for-age
SD: Standard deviation
TEE: Total energy expenditure
USDA: United States Department of Agriculture
WHO: World Health Organization
